# Sustained Effect of Music Training on the Enhancement of Executive Function in Preschool Children

**DOI:** 10.3389/fpsyg.2019.01910

**Published:** 2019-08-22

**Authors:** Yue Shen, Yishan Lin, Songhan Liu, Lele Fang, Ge Liu

**Affiliations:** ^1^School of Psychology, Liaoning Normal University, Dalian, China; ^2^Liaoning Collaborative Innovation Center of Children and Adolescents Healthy Personality Assessment and Cultivation, Dalian, China; ^3^The Forth Kindergarten of Shahekou, Dalian, China

**Keywords:** music, training, executive function, preschool children, sustained effect

## Abstract

Musical training is an enrichment activity involving multiple senses, including auditory, visual, somatosensorial, attention, memory, and executive function (EF), all of which are related to cognition. This study examined whether musical training enhances EF in preschool children who had not undergone previous systematic music learning. This study also explored the after-effects 12 weeks after cessation of musical training. Participants were 61 preschool children from a university-affiliated kindergarten in North China. The experimental group underwent 12 weeks of integrated musical training (i.e., music theory, singing, dancing, and role-playing), while the control group performed typical daily classroom activities. The three components (inhibitory control, working memory, cognitive flexibility) of executive functions were evaluated using the Day/Night Stroop, Dimensional Change Card Sort, Dot Matrix Test, and Backward Digit Span Task. In Experiment 1, EFs were tested twice-before (T1) and after (T2) the music training. The results showed that children’s EFs could be promoted by musical training. In addition, EFs were tested again 12 weeks later after the end of the intervention (T3) in Experiment 2. We discovered that integrated musical training demonstrated a sustained promotion effect.

## Introduction

### Executive Function and Training

Executive Function (EF) refers to a family of top-down mental processes necessary for concentration, specifically when relying on instinct, intuition, or automatic processing would be ill-advised, insufficient, or impossible (Miller and Cohen, 2001; Espy, 2004; Burgess and Simons, 2005). The division of EF dimensions by various researchers is still controversial. For instance, Miyake et al. (2000) focused on the shifting of mental sets, monitoring/updating of working memory representations, and inhibition of prepotent responses as the three subcomponents of EF; while Garon et al. (2008) labeled EF as inhibition, shifting, and working memory. However, the current study follows the definitions proposed by Diamond (2013), who considered EF to consist of inhibitory control, working memory, and cognitive flexibility. Specifically, these three components were defined as (1) Inhibitory control: the aspect of inhibitory control that involves resisting temptations and not acting impulsively or prematurely; (2) Working memory (WM): holding information in mind and mentally working with it (e.g., relating one thing to another, using information to solve a problem); and (3) Cognitive flexibility: changing perspectives or approaches to a problem, flexibly adjusting to new demands, rules, or priorities (as in switching between tasks) (Diamond, 2013, p. 137).

Executive Function efficiency is an important factor in ensuring physical/mental health, a key predictor of academic/career achievements, and also it plays a vital role in cognitive, social, and psychological development (Baler and Volkow, 2006; Brown and Landgraf, 2010; Morrison et al., 2010; Miller, 2011). EF is incredibly plastic, which can be improved throughout the lifespan. However, the plasticity of EF tends to show a gradual downward trend with aging (Fernández-Ballesteros et al., 2003; Lustig et al., 2009). Therefore, in the early stages of child development, targeted training of EF can allow cognitive ability, as well as physical and mental development, to reach a higher level.

To date, researchers have used interventions such as sports, meditation, and gaming to promote children’s EF development (Lakes and Hoyt, 2004; Winsler et al., 2011; Becker et al., 2014; Razza et al., 2015) (Table 1). For instance, Lakes and Hoyt (2004) randomly divided 207 children into experimental and control groups. Experimental group underwent 3 months of martial arts training, while the control group underwent 3 months of traditional sports training. Following training, children in the experimental group exhibited improved cognitive self-regulation, affective self-regulation, prosocial behavior, classroom conduct, and performance on a mental math test than children of the control group. Additionally, Razza et al. (2015) divided 29 children, aged three to five, into experimental and control groups, and then trained the experimental group in yoga based meditation. Children were assessed via behavior questionnaire, the toy wait task, and the pencil-tapping task, both before and after training. Results showed a remarkable increase in the experimental group’s inhibition ability. Further, Becker et al. (2014) used brief sessions to develop EF of children, aged four to six. Following intervention, children’s inhibition ability was generally increased, as well as their ability in reading and mathematics. Winsler et al. (2011) conducted a combination of sound and behavioral musical training for 89 children, between the ages of three and five. Using the gift delay task, dragon/bear game, straight-line task, and other behavioral inhibition experiments, the researchers examined whether the experimental group could suppress its dominant response after musical training. Results showed that the scores of the dominant response of the children who had undergone musical training were significantly higher than those of the control group, and the children who had undergone musical training demonstrated a significant increase in self-speaking in the selective attention task.

**TABLE 1:** A synthetic table of EFs.

**The type of training**	**The effects on the specific abilities of EFs**	**References**
Computerized training	Working memory	Klingberg et al., 2005; Holmes et al., 2009; Johnstone et al., 2010; Bergman Nutley et al., 2011
Arts training (e.g., martial arts, mindfulness practices, yoga)	Inhibitory control Working memory	Manjunath and Telles, 2001; Lakes and Hoyt, 2004; Flook et al., 2010; Zelazo and Lyons, 2012; Razza et al., 2015
Sports	Cognitive flexibility, Working memory, Inhibitory control	Tuckman and Hinkle, 1986; Sarnthein et al., 1997; Lakes and Hoyt, 2004; Bergman Nutley et al., 2011; Davis et al., 2011; Kamijo et al., 2011
Music training	Cognitive flexibility, Working memory, Inhibitory control	Degé et al., 2011; Moreno et al., 2011; Winsler et al., 2011
Task training (e.g., the delay of gratification task: flanker, go/no-go)	Inhibitory control	Binder et al., 2000; Traverso et al., 2015
Add-Ons to Classroom Curricula (e.g., Promoting Alternative Thinking Strategies, the Chicago School Readiness Project)	Inhibitory control Cognitive flexibility	Kusché et al., 1993; Webster-Stratton and Reid, 2004; Riggs et al., 2006; Raver et al., 2008; Zhai et al., 2011

According to a comprehensive meta-analysis of EF training programs (Diamond and Ling, 2016), there are several ways to improve EF. These include computerized cognitive training, Montessori-based school curricula, martial arts, and yoga. To be successful, key elements, such as high-quality activity presentation, adequate practice time, and constantly challenged EF should be included in the training programs (Diamond and Ling, 2016). Diamond and Lee (2011) concluded that the best approaches to improve EF and school outcomes would likely be those that (i) engage students’ passionate interests, bringing them joy and pride; (ii) address stressors in students’ lives, attempting to resolve external causes, and to strengthen calmer, healthier responses; (iii) have students vigorously exercise; and (iv) give students a sense of belonging and social acceptance, in addition to giving students opportunities to repeatedly practice EF at progressively advancing levels.

### Musical Training

At present, there are many training methods to improve EF, but musical training has characteristics of a wide transfer effect, challenging training content, and time-consuming practice. In musical training, children train independently, showing interest, motivation, and pleasure in training. This makes musical training an appropriate method for promoting children’s EF. Compared to previous studies, musical training is more accordant with the characteristics of promoting EF development proposed by Diamond and Ling (2016). During the process of musical training, individuals need to pay appropriate attention to information from each sensory channel, switch between different sensory simulations in real time, integrate information from multiple sensory channels, and save this information to working memory so that it available for recall at any time, all while restraining interference of other external competitive stimuli (Moradzadeh et al., 2014; Sato et al., 2015; Slevc et al., 2016). Furthermore, not only is musical training a comprehensive type of training, which is more complex than other types of general cognitive training, but it is also typically considered more interesting and attractive than other types of training. Additionally, if the individual is committed to training, it makes him/her less sensitive to the cognitive load, and the direct benefits gained from training further enhances the individual’s intrinsic motivation to learn (Okada, 2016). Therefore, long-term, intensive musical training could improve EF, both comprehensively and effectively (Seinfeld et al., 2013; Slevc et al., 2016).

Investigation of differences in brain structure and function between musicians and non-musicians has become an effective way to explore brain plasticity (Schlaug, 2003; Strait and Kraus, 2014). The journal *Nature* reported that the spatial reasoning of college students improved when preceded by 10 min of listening to Mozart’s Sonata for Two Pianos in D major, K. 488 (Rauscher et al., 1993). The “Mozart Effect” not only triggered interest in musical training, but also received great attention from the public. Increasing numbers of children and adolescents started learning music. With deepening research, the transfer effect of musical training has been verified. For example, musical training can improve children’s intelligence quotient (IQ), self-control, reading ability, mathematics ability, and memory (Schellenberg, 2005).

It is not clear which mechanisms of musical training affect cognitive ability, but many researchers believe that the transfer effect of musical training involves EF (Hannon and Trainor, 2007; Schellenberg et al., 2008; Jäncke, 2009; Moreno et al., 2014; Moreno and Farzan, 2015; Saarikivi et al., 2016; Sala and Gobet, 2017). Early researchers argued that EF played a mediating role in IQ improvement; however, this mediating role was controversial (Degé et al., 2011; Schellenberg and Winner, 2011). Currently, researchers explain the relationship between musical training and EF from two viewpoints. Some researchers believe that the improvement in EF is the far transfer effect of musical training. For instance, Miendlarzewska and Trost (2014) conducted a meta-analysis of the far- and near-transfer effects of musical training. They concluded that factors affecting musical training include genetics, the time when musical training begins, the motivation to learn music, parents, teachers, the process of social development, and the emotional experience brought by music. The near-transfer effects of musical training are reflected in auditory skills, motor skills, and time perception ability, while the far-transfer effects of music are reflected in EF, intelligence, auditory perception, reading, verbal memory, and social interaction ability. Moreno and Bidelman (2014) proposed that the cognitive transfer effects of musical training can be described as a multidimensional continuum. They used the two dimensions of “near/far” and “sensory/cognition” to explain the transfer effects of musical training. For example, the improvement of auditory perception by musical training is a “near/sensory” transfer, the improvement of complex sound processing ability by musical training is a “near/cognitive” transfer, the improvement of speech and language coding ability in musical training is “far/sensory” transfer, and the effects on auditory recognition of patterns and phonological awareness are “far/cognitive” transfer. The two-dimensional transfer model is based on the hypothesis that the uniqueness of musical training itself can promote improvement of the musician’s EF, and this EF improvement is the basis of other transfer effects of musical training.

In the process of learning music, it is necessary to maintain a high degree of self-control, attention, and memory (Moreno et al., 2011). Moreover, musicians are constantly monitoring and regulating their behaviors. This requires them to sustain attention for long periods of time, ultimately resulting in improvement of their listening and speech skills. According to the OPERA hypothesis (Patel, 2011, 2012), music enhances auditory processing in ways that are relevant to speech when five conditions are met; specifically, overlap, precision, emotion, repetition, and attention. Patel (2014) also considered that music and speech shared perceptual and cognitive processes, which was anatomical overlap in brain networks (e.g., waveform periodicity and amplitude envelope). Speech processing benefits by music learning, which naturally improves due to its more precise requirement for sound representation, stronger emotion, repetition, and high concentration of attention.

In other words, the transfer effect is determined by the extent to which musical training contributes to EF (White et al., 2013). Researchers holding either perspective believe that musical training can improve EF. The mechanism responsible for the musical training transfer effect can be explained to a certain extent by the complementary dimensions expressed by Moreno, while the musical training transfer effect model emphasizes the phenomenon of the “near-far” transfer effect of musical training.

### Musical Training and EF in Developmental Research

The developmental time point in which a person begins receiving musical training is also a key factor in how music affects individuals. Research suggests that better results are achieved if musical training begins in childhood, rather than adulthood; a finding that has been unanimously recognized (Schlaug et al., 1995; Baharloo et al., 1998; Lee et al., 2003). For instance, Baharloo et al. (1998) performed absolute pitch tests in 691 adult musicians and found that 92 showed excellent absolute pitch ability. Of these, 78% began learning music before the age of six.

In 1995, Schlaug et al. studied the neuroanatomical differences between musicians and non-musicians. They found that musicians’ corpora callosa were larger than those of the non-musicians. The corpus callosum is a transverse nerve fiber bundle that connects the two hemispheres of the brain. Maturity of the corpus callosum’s structure and function likely occurs in late childhood to early adolescence; this period corresponds with the development of motor control and coordinated motion (Schlaug, 2003). A study by Lee et al. (2003) reported that the corpus callosum of males who performed rigorous musical training (piano and string practice) in early childhood were larger than those of non-musical trainers. Such evidence was only found in male participants who studied music before the age of seven. There are two reasons why the corpus callosum of a musician is larger. First of all, no matter which instrument you are learning, you need to coordinate the right and left hands. This makes the movement areas of the left and right brains more developed. Furthermore, long-term musical training can promote the exchange of information between the two hemispheres of the brain, enabling more cooperation between them. Second, early musical training is carried out during a period of rapid development of the corpus callosum; in other words, early initiation of musical training plays an important role in promoting the development and maturation of the corpus callosum. Watanabe et al. (2006) found that adult musicians who began to receive musical training before the age of seven performed significantly better in time series tasks than adult musicians who began to receive musical training after the age of seven. Therefore, it was concluded that in early life there is a critical period during which music promotes the development of brain-related functions. Other researchers have shown that the improvement observed in the auditory cortex and neurophysiological function of musicians is positively correlated with the time of continuous training and negatively correlated with the age at which musical training begins (Zendel and Alain, 2013). That is, the longer one has practiced music, and the earlier musical training begins, the greater the likelihood that the cerebral cortex and cognition system will change in response to musical training.

Although there is a dearth of pre-/post-test experiments, a large body of evidence exists suggesting that children’s EF can be improved after a certain period of musical training. For example, Moreno et al. (2011) conducted a four-week structured musical training for 32 children aged four to six; the training included information regarding rhythm, beat, melody, sound, and basic music theory. Results showed that after the 4 weeks of musical training, the experimental group performed better on the control task than the control group. Bowmer et al. (2018) used a two-phase experimental design to investigate the effect of weekly musical training on the EF abilities of children aged three to four. Participants were divided into groups A, B, and C. In Phase 1, Group A took part in eight weekly music lessons, which were provided by a specialized music teacher. While Groups B and C engaged in free play. Results of this Phase showed that Group A’s planning and inhibition skills were improved. In Phase 2, Group A continued eight additional weeks of music curricula, and Group B attended the same eight-week music curricula attended by Group A in Phase 1. Conversely, Group C took part in an art intervention. The result showed that, at the end of the two experimental phases, the children who participated in musical training demonstrated significant improvement in EF. A separate study examined the effects of 6 weeks of musical training or Lego ^®^ construction in 34 randomly assigned preschool children, aged four to five. All the participants attended 45 min of training twice a week. The music program was focused on bimanual gross motor behavior, creativity, and vocal development of inhibition ability. Results showed the music group demonstrated fewer errors on a visual-motor inhibition task following training when compared to the Lego^®^ group, despite between group differences not being observed (Bugos and DeMarie, 2017).

In the short-term, simply listening to music and structured musical training are representative of interventions studied in the field. Structured musical training generally includes the learning of musical knowledge (e.g., identifying notes, rhythm and beats) and the learning of musical skills (e.g., vocal and keyboard skills; Moreno et al., 2011; Winsler et al., 2011). Winsler et al. (2011) pointed out that structured musical training has a larger impact on subjects. Moreno et al. (2011) conducted musical training with young children, ranging from one month to one year old. The researchers found that musical training resulted in positive changes in children’s brain structure, inhibitory control, increased attention, and enhanced creativity (Fujioka et al., 2006; Schellenberg et al., 2007; Moreno et al., 2011). Other researchers conducted a five-day piano exercise for individuals with no prior formal music learning experience. After the exercise, participants’ motor cortex, finger flexors, and extensors had increased in size (Pascual-Leone et al., 1995). While a large number of studies have shown effects of musical training, regardless of training duration, these effects appear to be short lived, and have not been well-studied.

In the present study, we designed two experiments, based on the conceptualizations of EF proposed by Diamond (2013), to explore the impact of musical training on children’s EF. Experiment 1 used integrated musical training to conduct a 12-week program for children who had not received any prior musical training (the mean age was four). We explored the promotion of musical training on three major subcomponents of children’s EF: inhibitory control, working memory, and cognitive flexibility. It was necessary to comprehensively investigate the influence of musical training on all three of these subcomponents. The EFs were tested at two points: before the music training (T1), immediately after the end of the training (T2). In Experiment 2, in order to explore the after-effects of integrated musical training, the children in the musical training and control groups were assessed 12 weeks after the experiment (T3). We hypothesized that (1) inhibitory control, working memory, and cognitive flexibility would be enhanced in preschoolers by integrated musical training (i.e., music theory, singing, dancing, and role-playing); and (2) 12 weeks after cessation of training, the after-effects of integrated musical training on inhibitory control, working memory, and cognitive flexibility would persist.

This study is different from previous studies in several ways. (1) In order to understand the level of musical development and preferences of children, aged three to six, we interviewed professional music teachers who worked in kindergartens. We also referred to previous research and designed musical training curricula (e.g., rhythm, pitch, melody, voice, and basic musical concepts) suitable for 4-year-old children. (2) Although the structure of EF is still debated, this study was based on the view put forth by Diamond (2013). Furthermore, previous studies have primarily focused on one component of EF (e.g., Moreno et al., 2011). The current study examined three components of EF. (3) According to Diamond and Ling (2016), effective training of EF requires cognitive components. We applied cognitive components within the contents of musical training protocol (e.g., children needed to understand the rhythm rules to correctly stay in time with the music). (4) The contents of the musical training protocol designed within this study was closely related to the three components of EF. It also has a clear training purpose and is easily operated. (5) Compared to previous research, we examined the after-effects of the training curricula 12 weeks later.

## Experiment 1

### Materials and Methods

#### Participants

The effect of music training is normally influenced by some variables, such as social background of participants. In order to maintain the homogeneity of the variables (kindergarten living environment, daily schedule, daily activities of kindergartens, etc.), we selected two classes of children (average age of 4) in the same university-affiliated kindergarten in northern China: one for the music training group and the other for the control group (Table 2). This kindergarten is a public kindergarten and nursery fee is 1200RMB per month, and the enrollment is citywide. Even it is affiliated to the university, it is as same as other public kindergartens in this city. The experiment was divided into two groups. Three measurements were made. The number of participates was 29 in each group, which met the criterion of G-power test. There are 30–35 children in every class of public kindergarten in this city. In order to ensure ecological validity and control the interference of irrelevant variables, two natural classes were selected.

**TABLE 2:** Mean age and gender of participant groups.

	**Total number of participants**	**Male**	**Female**	**Average age (months)**	**Standard deviation**
Music training group	30	20	10	51.39	4.27
Control group	31	18	13	50.35	3.38

The age difference between the music training group and the control group was not significant *F* = 3.799 (*p* > 0.05). All participants were healthy, well-being, and had no formal music learning experience. Parents did not have a background in music-related occupations. The average annual household income was 120,000–180,000 yuan (RMB). Which was middle-income family compared to that in Liaoning Province. This study was approved by the local ethics committees of Liaoning Normal University. Written informed consent had been obtained from the parents/legal guardians of all participants. All preschool children participants were volunteered to join the experiments, and informed consents were signed by their legal guardians.

#### Training Curricula

The children engaged in the training programs in one team of 45 min each (10 min for organization and 35 min of training), 5 days a week, for 12 weeks (150 min per week). The music training was based on a combination of motor, perceptual, and cognitive tasks, including training in rhythm, pitch, melody, voice, and basic musical concepts.

For short-term music training participants, the following two methods are more representative. One is to sit and listen to music and experience the Mozart Effect; this could include letting college students listen to Mozart’s double piano sonatas. The other method allows participants to perform structured music training, which generally includes the learning of music knowledge (identifying notes, rhythm, beats, etc.) and the learning of musical skills, such as vocal or keyboard skills, conducted a 4-week structured music training for 32 children aged 4 to 6 years; the training included topics on rhythm, beat, melody, sound, and basic music theory. The research results showed that after the 4 weeks of music training, the experimental group performed better on the control task than the control group (Moreno et al., 2011).

The selected songs in this experiment are from the *John Thomson’s Modern Course for the Piano* (WILLIS, Shanghai Music Publishing House), and the researchers created their own tracks based on the teaching content. To reduce the cognitive load of children, the music training tracks were used in a multi-purpose way. In other words, we could use one track to train participants in singing, dancing, role-playing, and so on. The music training program of this research included two sections (1–4 weeks; 5–12 weeks). We used the second- (Table 3) and tenth-week (Table 4) training programs to illustrate the relationship between executive function and the content of training in different stages.

**TABLE 3:** Examples of music activities included in the intervention and associated areas of EFs (Second week).

	**Example musical training (Policeman)**	**Associated area of EFs**
Day 1	Clef and Scale: Identify treble and C major, a minor scale	Working memory, cognitive flexibility
Day 2	Time signature: Listen to 3/4 beats, and be able to follow them	Working memory, cognitive flexibility
Day 3	Termination mark: Identify the termination token, and stop when you see the termination token	Working memory, inhibitory control
Day 4	Strong and weak symbol: Identify strong and weak marks(F/P), which can control sound according to strong and weak marks	Working memory, cognitive flexibility
Day 5	Repeated mark: Identifying repeated marks, and repeat then according to repeated mark indications	Working memory, inhibitory control, cognitive flexibility

**TABLE 4:** Examples of music activities included in the intervention and associated areas of EFs (Tenth week).

	**Example musical training (Frog chorus)**	**Associated area of EFs**
Day 1	Solo: A young children sings alone, keeping the pitch and rhythm correct	Working memory, inhibitory control, Cognitive flexibility
Day 2	Rotate in turn: Different children rotate and alternately sing the same song	Working memory, inhibitory control, cognitive flexibility
Day 3	Sing in silent: Sing without sound	Working memory, inhibitory control
Day 4	Role performance: Rhythms was divided into two parts (young frog and old frog) according to pitch, the children who acted as young frog started to sing when the young frog rhythms appeared, the others acting as the old frog should wait quietly, and vice versa.	Working memory, inhibitory control
Day 5	Dance: Rhythmic action and action combinations, including clapping, nodding, stamping feet, and so on.	Working memory, Inhibitory control, cognitive flexibility

The time of effective music training for children was 35 min every day. The order of each music activity was fixed. The melody was active and lively. The difficulty of weekly training is from simple to complex. It gradually increased the difficulty and the training purpose was clear. The purpose of the selected track was clear, the difficulty of music rules gradually increased, the melody was active and lively, the music was mainly about animals and daily life (themes that the children loved), and the rhythm was selected as 2/4 beats and 3/4 beats, emphasizing the enthusiasm, regularity, integration, cheerfulness, and playfulness of the music training. The content was designed to encourage the children to actively participate and experience pleasure in participating. The goal was for children to then follow the rules of music, suppress impulsive behavior, recognize and memorize music symbols, and flexibly use music symbols. All the activities of this music training program were carried out by the same Master of Musicology. This person had a solid theoretical foundation of music teaching, practical experience in early childhood music teaching, relevant knowledge of development and educational psychology, and experimental research experience in kindergarten settings. Furthermore, they could better implement the guiding ideology of music training activities and mobilize the enthusiasm of young children to participate in music training than the experimenter, who only had a psychological background.

The musical training consisted of two parts (see Tables 3, 4):

• Weeks 1–4: musical theory
• Weeks 5–12: singing, dancing, and role-playing

#### Stimuli and Procedures

The executive functions of the children from all groups were assessed twice-before (T1) and after (T2) the musical training.

#### Day/Night Stroop

The task was based on study Gerstadt et al. (1994). The experiment used 16 test cards, 20 cm long and 13 cm wide. Half of the cards were painted with a white sun and half with a black moon and stars. The experimental task was divided into two phases. During the practice phase, the experimenter first presented a white card with a bright sun to the child and told the child to say “day” when the child sees the white card; then the experimenter presented a black card with the moon and stars to the child and asked the child to say “night” when the child sees the black card. Then, the experimenter showed the subject a white card. If the subject responded correctly, the experimenter praised the child and proceeded to a practical trial with the black card. If the subject responded correctly to the black card, the experimenter praised the child. If the subject responded incorrectly or did not respond at all on either of these trials, the experimenter immediately reminded the subjected of both rules beginning with the card that the child had identified incorrectly.

During the formal experiment, the experimenter presented the opposite condition to the child, this time no feedback was given. The experimenter presented a white card to the child and told the child to say “night” when the child sees the white card. Then the experimenter presented a black card to the child and asked the child to say “day” when the child sees the black card.

The experiment was performed 32 times, and the cards were presented according to a pseudo-random sequence. We recorded the number of times the participant gave the correct “day” or “night” response. In this task, the Cronbach’s Alpha was 0.60.

#### Dimensional Change Card Sort

Before the formal experiment, we conducted a pre-experiment on 3–5-year-old children in other classes of the kindergarten. The pre-experiment results showed that the three-stage experiment was difficult, the cognitive load of the child was too heavy, and the experiment time was long. Therefore, we only carried out the two-stage experiment task, and in the formal experiment, the two-stage experiment task did not reach the ceiling-effect.

The experiment used 16 cards and 2 wooden plates. The cards were 20 cm long and 13 cm wide, each wooden plate is 11.5 cm long, 9.5 cm wide and 2 cm deep. Children had to sort the cards according to a rule involving either color or shape. They were shown cards with boats or rabbits on them, either blue or red in color. The target cards were fixed to the back of each wooden plate, one showing the image of a blue rabbit, and the other a red boat. The experimenter pointed and verbally named the two target cards. In the pre-switch phase, children were asked to sort six cards according to their color, after two demonstrations given by the experimenter. Cards were presented to the child in a pseudo-random order. In the post-switch phase, children were asked to sort the cards by shape. The test was scored according to the number of correct cards. Both the pre-switch phase and post-switch phase tasks were each scored once (Zelazo et al., 1996; Zelazo, 2006). In this task, the Cronbach’s Alpha was 0.61.

#### Dot Matrix Test

The experimental materials were 16 test cards with red, green, and blue dots. The cards were 20 cm long and 13 cm wide. The dots on the cards were randomly arranged. During the test, children were instructed to count the number of red spots on the card presented. After an initial practice session, children were presented with two cards that were facedown on the table. The experimenter then turned the first card faceup; after the child counted the red spots, this card was turned facedown and the second card was turned faceup. After counting, this card was turned facedown. The experimenter pointed to the first card and then the second, asking the child to recall the number of spots counted on each card. Administration of the test continued until the child made errors on both attempts at a particular span length. This span was recorded as the maximum number of counts recalled in the correct serial order (Towse and Hitch, 1995; Bull and Scerif, 2001). In this task, the Cronbach’s Alpha was 0.86.

#### Backward Digit Span Task

Before the formal experiment, we conducted a pre-experiment on 3–5-year-old children in other classes of the kindergarten, and finally selected 1–4 digits as the numerical range of the Backward digit span task.

The experimental materials for this task were the numbers 0–9. The experiment was divided into two phases. In the practice phase, the experimenter said “1, 2” and told the child to say “2, 1,” i.e., reciting the numbers backward. In the formal experiment, the experimenter randomly selected two numbers from 0 to 9, and then let the child say the numbers backward. If the child failed, they scored 1 point. If the child was successful, they scored 2 points, and the experimenter continued on, saying 3 digits. If the child successfully recited the 3 digits backward, they scored 3 points, and then the experimenter moved up to 4 digits. The maximum number of digits used was 4 (Carlson et al., 2002). In this task, the Cronbach’s Alpha was 0.79.

#### Statistical Analysis

In this experiment, the scores of the 4 tasks were the dependent variables, and the time points (pre-test vs. post-test) and the groups (the experimental group vs. the control group) were independent variables. The experimental design was a 2 (time points: pre-test vs. post-test) × 2 (groups: the experimental group vs. the control group) two-way repeated measures ANOVA, in which the time points were the intra-group variables, and the groups were the inter-group variables. The analysis of variance mainly examined the interaction between time points and groups. In the control of unrelated variables, we took the following measures. First, we conducted a survey of two classes of children during the pre-test to ensure that there were no additional music training activities outside the kindergarten environment. Second, we applied a homogeneity test on the pre-test group to ensure both groups’ developmental level of executive function. While the experimental group underwent music training, the children in the control group engaged in free play. In addition, the daily activities were the same. Uniform requirements were imposed on all teachers, and teachers were not allowed to impose additional activities on the children. Fourth, we informed parents to control additional music training activities.

### Results

The scores of children’s executive function tasks before and after music training are shown in Table 5.

**TABLE 5:** The executive function task data of the experimental group and the control group.

	**Music Training Group**		**Control Group**	
	**(*n* = 30)**		**(*n* = 31)**	
	**T1 (*M SD*)**	**T2 (*M SD*)**	**T1 (*M SD*)**	**T2 (*M SD*)**
Day/Night Stroop	12.97(4.62)	23.10(1.16)	12.00(6.08)	18.52(4.70)
DCCS	12.50(2.93)	15.53(1.814)	12.55(2.71)	13.48(2.49)
Dot Matrix Test	0.83(0.87)	1.83(0.75)	0.77(0.99)	0.84(0.86)
Backward Digit Span Task	7.30(2.09)	8.77(1.83)	6.52(2.31)	6.90(2.31)
				

In order to better control the experimental variables, we performed statistics on the pre-test results. The results showed that there was no significant difference in the scores of the 4 tasks [Day/Night Stroop, Dimensional Change Card Sort (DCCS), Dot Matrix Test, Backward Digit Span Task] between the experimental group and the control group (*t*(59) = 0.697, –0.67, 1.390, 0.247, *p* > 0.05), thus indicating that the two groups of children were homogeneous in levels of executive function.

The results of the 2 (time points: T1 vs. T2) × 2 (groups: the experimental group vs. the control group) two-way repeated measures ANOVAs are shown in Table 6.

**TABLE 6:** Analysis of variance analysis before and after music training.

**Task**	**Source**	***df***	***MS***	***F***	**η^2^_p_**
Day/Night Stroop	Time	1	2113.117	133.398^∗∗∗^	0.693
	Group	1	234.851	9.257^∗∗^	0.136
	Time × Group	1	99.740	6.296^∗∗^	0.096
DCCS	Time	1	120.73	26.996^∗∗∗^	0.314
	Group	1	30.525	3.691^Δ^	0.59
	Time × Group	1	33.548	7.543^∗∗^	0.113
Dot Matrix Test	Time	1	26.196	19.221^∗∗∗^	0.246
	Group	1	53.424	7.370^∗∗^	0.111
	Time × Group	1	8.884	6.519^∗∗^	0.099
Backward Digit Span Task	Time	1	8.638	23.234^∗∗∗^	0.283.
	Group	1	8.465	7.343^∗∗^	0.111
	Time × Group	1	6.671	17.943^∗∗∗^	0.233

In the Day/Night Stroop, the interaction between the time points and groups was significant [*F*(1,60) = 6.296, *p* < 0.01, η^2^ = 0.096]. In the *post hoc* test, the difference between the experimental and control groups was significant (*t* = 5.19, *p* < 0.001). The difference between the T1 and T2 results of the experimental group was significant (*t* = –11.45, *p* < 0.001), and the difference between the T1 and T2 results of the control group was significant (*t* = –5.77, *p* < 0.001).

In DCCS, the interaction between time points and groups was significant [*F*(1,60) = 7.543, *p* < 0.01, η^2^ = 0.113]. In the *post hoc* test, the difference between the experimental and the control groups was significant (*t* = 3.67, *p* < 0.001). The difference between the T1 and T2 results of the experimental group was significant (*t* = –5.56, *p* < 0.001), and the difference between the T1 and T2 results of the control group was not significant (*t* = –1.75, *p* > 0.05).

In the Dot Matrix Test, the interaction between time points and groups was significant [*F*(1,60) = 6.519, *p* < 0.01, η^2^ = 0.099]. In the *post hoc* test, the difference between the experimental and the control groups was significant (*t* = 3.75, *p* < 0.001). The difference between the T1 and T2 results of the experimental group was significant (*t* = –4.52, *p* < 0.001), and the difference between the T1 and T2 results of the control group was not significant (*t* = –1.42, *p* > 0.05).

In the Backward Digit Span Task, the interaction between the time points and groups was significant [*F*(1,60) = 17.943, *p* < 0.001, η^2^ = 0.233]. In the *post hoc* test, the difference between the experimental and the control groups was significant (*t* = 4.82, *p* < 0.001). The difference between the T1 and T2 results of the experimental group was significant (*t* = –5.79, *p* < 0.001), and the difference between the T1 and T2 results of the control group was not significant (*t* = –0.47, *p* > 0.05).

In order to investigate if there were any difference between the gains of experimental group and control group before and after music training, we took post-assessment scores T2 to minus pre-assessment scores T1, and did independent sample *T*-test to control the impact of T1 on experiments results. The results were shown in Table 7. The results indicated that the gains from four executive function experiments had significant differences between the two groups, which demonstrated great impact of music training on experimental group.

**TABLE 7:** *T*-test analysis of the gain (T2-T1).

	**Groups**	***N***	***M***	***SD***	***t***	***df***
Day/Night Stroop	Experimental group	30	10.133	4.84756	2.509^∗^	59
	Control group	31	6.516	6.29217		
DCCS	Experimental group	30	3.033	2.98829	2.746^∗∗^	59
	Control group	31	0.935	2.97697		
Dot Matrix Test	Experimental group	30	1.466	1.77596	2.553^∗^	59
	Control group	31	0.387	1.52047		
Backward digit span task	Experimental group	30	1.000	0.94686	4.236^∗∗∗^	59
	Control group	31	0.064	0.77182		

### Discussion

The results of Experiment 1 showed that 12 weeks of integrated musical training could promote the development of children’s EF, a finding that was consistent with the results of previous research (Moreno et al., 2011; Winsler et al., 2011). This experiment was a classic design, in which we randomly chose two classes, with roughly the same number of students, to participate (i.e., experimental and control groups). In addition to the daily musical training, the experimental group still participated in everyday activities typically performed in kindergarten. The musical training duration fit within a natural semester, which was slightly different from what has been reported in previous research. In studies with random recruitment, participants’ interest in musical training was less likely (Corrigall et al., 2013). Furthermore, with a long duration of musical training, participants had a halfway point from which they could exit. For example, Schellenberg (2004) conducted a one-year musical training protocol for 144 children; of which, 12 participants withdrew. The kindergartens selected in the present study were university-affiliated kindergartens. The children’s parents had a high degree of matching in terms of education level and family annual income. No children withdrew throughout the 12-week musical training program. The musical training protocol utilized in the current study was closer to the daily teaching activities of young children, thus laying the foundation for the promotion of musical training.

A large number of related studies have shown that short-term or long-term musical training can affect the brain structure, function, and cognitive level of those involved in the training (Rauscher et al., 1993; Schellenberg, 2006; Moreno et al., 2011; Schellenberg and Winner, 2011). However, most previous studies did not track the effect of musical training. Thus, it is still not clear whether musical training can have long-term effects in children. In Experiment 2, the children in the musical training experimental group and the control group were followed up at 12 weeks following cessation of the musical training program. Thus, the current study examined both the development of EF of the two groups of children and explored the duration of the musical training transfer effect.

## Experiment 2

### Materials and Methods

#### Participants

The participants were the same as those in Experiment 1.

#### Stimuli and Procedures

The stimuli were the same as those in Experiment 1.

In Experiment 2, we used the same performance test materials to conduct after-effects tests on the children in the music training experimental group and the control group at T3 (12 weeks after Experiment 1). Leading up to this period, the researchers asked the parents of the participating children to ensure their children did not have any in-school or extra-curricular music training activities.

#### Statistical Analysis

In this experiment, the scores of the 4 tasks were the dependent variables, and the time points (T2 vs. T3) and the groups (the experimental group vs. the control group) were independent variables. The experimental design was a 2 (time points: T2 vs. T3) × 2 (groups: the experimental group vs. the control group) two-way repeated measures ANOVAs, in which the time points were the intra-group variables and the groups were the inter-group variables. The analysis of variance mainly examined the interaction between time points and groups.

### Results

The results of the after-effect of executive function tasks of each group of children in Experiment 2 at T3 are shown in Table 8.

**TABLE 8:** After-effect description statistics at T3.

	**Experimental group**		**Control group**	
	**(*n* = 30)**		**(*n* = 31)**	
**Task**	***M***	***SD***	***M***	***SD***
Day/Night Stroop	21.73	1.143	19.19	2.315
DCCS	15.37	1.474	14.42	1.432
Dot Matrix Test	8.87	2.063	7.84	1.934
Backward Digit Span Task	1.63	0.718	1.10	0.597

We used the 4 experimental scores as the dependent variables. The time points (T2 vs. T3) and the groups (the experimental group vs. the control group) were independent variables. The results of the 2 time points (T2 vs. T3) × 2 groups (the experimental group vs. the control group) two-way repeated-measure ANOVAs are shown in Table 9.

**TABLE 9:** Music training after-effect repeated measurement analysis of variance.

**Task**	**Source**	***df***	***MS***	***F***	**η^2^_p_**
Day/Night Stroop	Time	1	3.621	1.150	0.019
	Group	1	386.837	31.908^∗∗∗^	0.351
	Time × Group	1	31.851	10.110^∗∗^	0.146
DCCS	Time	1	4.506	2.556	0.042
	Group	1	68.459	13.388^∗∗^	0.185
	Time × Group	1	9.260	5.252^∗^	0.082
Dot Matrix Test	Time	1	8.174	6.671^∗^	0.102
	Group	1	63.729	9.755^∗∗^	0.142
	Time × Group	1	5.321	4.343^∗^	0.069
Backward Digit Span Task	Time	1	0.026	0.062	0.001
	Group	1	17.872	26.582^∗∗∗^	0.311
	Time × Group	1	1.599	3.873^Δ^	0.062

*PS: Time refers to the first measurement and second measurement; Group refers to the experimental group and the control group; ^∗^*p* < 0.05, ^∗∗^*p* < 0.01, ^∗∗∗^*p* < 0.001, ^Δ^*p* < 0.1.*

In the after-effects test of the Day/Night Stroop, the interaction between the time points and the groups was significant [*F*_(1,60)_ = 10.110, *p* < 0.01, η^2^ = 0.146]. The *post hoc* test showed that the results of the post-effects of the experimental group and the control group were significantly different (*t* = 5.404, *p* < 0.001), the difference between the two time points in the experimental group was significant (*t* = 5.646, *p* < 0.001), and the difference between the two time points in the control group was not significant (*t* = –1.153, *p* > 0.05).

In the after-effects test of the DCCS, the interaction between the time points and the groups was significant [*F*(1,60) = 5.252, *p* < 0.05, η^2^ = 0.082]. The *post hoc* test showed that the results of the post-effects of the experimental group and the control group were significantly different (*t* = 2.546, *p* < 0.05), the difference between the two time points in the experimental group was not significant (*t* = 0.556, *p* > 0.05), and the difference between the two time points in the control group was significant (*t* = –2.503, *p* < 0.05).

In the after-effects test of the Dot Matrix Test, the interaction between the time points and the groups was significant [*F*(1,60) = 4.343, *p* < 0.05, η^2^ = 0.069]. The *post hoc* test showed that the results of the post-effects of the experimental group and the control group were significantly different (*t* = 2.008, *p* < 0.05), the difference between the two time points in the experimental group was not significant (*t* = –0.356, *p* > 0.05), and the difference between the two time points in the control group was significant (*t* = –3.275, *p* < 0.001).

In the after-effects test of the Backward Digit Span Task, the interaction between the time points and the groups was significant [*F*(1,60) = 3.873, *p* < 0.1, η^2^ = 0.062]. The *post hoc* test showed that the results of the post-effects of the experimental group and the control group were significantly different (*t* = 3.176, *p* < 0.01), the difference between the two time points in the experimental group was not significant (*t* = 1.140, *p* > 0.05), and the difference between the two time points in the control group was not significant (*t* = –1.680, *p* > 0.05).

### Discussion

Twelve weeks following the cessation of musical training, we again tested the EF of the two groups of children and explored the duration of the transfer effect of musical training. Leading up to this period, the parents of the children who participated in the experiment were asked to ensure that their child avoided any additional musical training. Our results showed that in the absence of additional musical training in both groups, the scores of the EF tasks in the experimental group remained significantly higher than those in the control group. While scores on the Day/Night Stroop decreased significantly, scores on the other three tasks did not; thus, the effect of musical training had a sustained effect. Ling et al. (2016) claimed that the Day/Night Stroop involves the inhibition of dominant responses, which is different from other cognitive inhibition tasks. Especially, the aim of the Day/Night Stroop paradigm is focused on preschooler’s control over his/her daily lives (say “sun” to moon/star and “moon/star” to sun). Children were taught everyday knowledge in kindergartens, however, the results of Day/Night Stroop declined 12 weeks after musical training cessation.

Scores on the DCCS and the Dot Matrix Test in the control group increased significantly with age, while scores on the Day/Night Stroop and Digit Span Task did not. While this shows that EF improved due merely to the development of age and other factors, the effect of this natural improvement was still not as pronounced as that seen in the musical training group. The results of Experiment 2 showed that the transfer effect continues to play a role after cessation of musical training.

## General Discussion

As the number of EF intervention studies increase, it is progressively more important to explore the most appropriate method of increasing EF. According to Diamond and Ling (2016) meta-analysis of EF interventions, efficient intervention programs should have high-quality activities, adequate duration of practice, challenging content, involve cognitive components, and be able to be widely transferred. We referred to these opinions and designed an integrated musical training program. This research adopted the integrated musical training method, which utilizes daily music education and teaching activities. This approach not only enables children to recognize, memorize, and use music rules and symbols through the study of music theory, but also integrates music rules, symbols recognition, memory, and application through singing, rhythmic activities, role playing, and music appreciation.

In musical training, whether children can understand music rules, memorize, recognize music symbols, sing, and/or tap a beat according to music rules and music symbols is the key to exhibiting melody. A musical melody is composed of notes according to the rules of music writing and using music symbols. To accomplish this, children need to understand the rules of music, including the symbols, and must be able to sing or play musical melodies according to these rules. Moreover, carrying out musical training requires cooperation from many people, and involves, for example, alternate singing, a chorus, and part singing. Therefore, children need to conform to singing order, detect the singing order of other children, and supervise/regulate their performance appropriately. This process requires a high degree of restraint, control, working memory, and cognitive flexibility. For example, in the musical training called “Finding Notes,” children need to quickly and accurately recognize different types of notes according to rules and test instructions. With understanding of the rules of notes, children not only can quickly and accurately find the corresponding notes, but also point out the mistakes of others.

In musical training, it is also important to be able to suppress and adjust behavior according to changes in musical symbols. Silent singing means singing without sound. We used this form of “singing” to train the inhibitory control of young children. In the early stage of musical training, it was difficult for children to sing without sound, while with the understanding of “silent singing” rules, young children could gradually suppress their impulse to sing out loud. For example, when learning the basics of music, the child would learn to terminate singing at a particular mark. When this mark appeared in the music, the child could suppress their urge to continue singing, and stop singing. In the case of beat practice, the test would add a “decrescendo” or “crescendo” symbol to the exercise according to the change rule of the beat. The child must then suppress their dominant reaction and produce either fortissimo or pianissimo beats.

In addition, musical training activities, such as turn singing, choral singing, and voice-singing have numerous rules. We created a situation for young children to perform a role, which allowed the children to become interested in our training, and ultimately, the constraints of music rules. Improving EF can also be promoted. For example, in the musical training called “Frog Chorus,” children can freely choose to role-play as a small frog or an old frog. Different characters sing a different melody. When the little frog sings, the old frog wants to listen quietly. When the old frog sings, the little frog wants to listen quietly. After the little frog and the old frog sing, all the frogs sing together. In the course of this training, the children are very active. After practicing a few times, most of the children can follow the order of the characters singing. When they are not singing the melody, they wait quietly and pay attention. Over time, musical training with clear rules and full of gameplay gradually improves performance.

Intensity is an important factor in musical training. For the auditory cortex, the intensity of musical training is positively correlated with left transverse temporal gyrus volume (Gaser and Schlaug, 2003). Even when age, sex, and Raven’s reasoning test scores were matched, the density of the left transverse temporal gyrus increased with the increase in musical training intensity (James et al., 2014). This integrated musical training totaled 60 days. The average weekly training time was 150 min, and the training intensity was high. Regarding training the EF of infants, Diamond and Lee (2011) believe that EF training should be regular; carried out under the guidance of teachers; include a wide range of transfer functions; include universal, convenient operation; and involve repetitive exercises. Compared with other training methods, musical training has the characteristics of regularity, interest, a wide transfer effect, and repetition (Miendlarzewska and Trost, 2014). Diamond and Ling (2016) also believes that EF must be constantly challenged, activity presentation must be of high-quality, and participants must spend an adequate length of time practicing. Integrative musical training is complex, but music itself is artistic and interesting, which makes it attractive to children. During training, children are not aware of the cognitive load under which they are placed. At the same time, they benefit from the aesthetic and interesting qualities of music, which enhances intrinsic motivation. Although there are many training methods to improve children’s EF, the interestingness and regularity of musical training gives it unique advantages in promoting the development of children’s EF.

Although the role of musical training in promoting EF has been supported by empirical research, most of the previous studies on the subject have not tracked the effects of musical training; thus it is not clear how long the effects last. To address this gap in the research, we tracked the children who participated in Experiment 1. 12 weeks after the end of musical training (T3), the EF level of the two groups of children was tested to explore the duration of the transfer effect of musical training. The results showed that scores in three major subcomponents of EF in the control group showed significant improvement, while the scores of the children in the musical training group slightly decreased; however, the level of EF in children in the musical training group was still significantly higher than that of children in the control group. This result shows that after musical training (T3), the transfer effect still plays a role. In a return visit to the teachers and parents of the children tested, we found that the children in the musical training group always thoroughly enjoyed music. These teachers and parents stated that many of the children practiced the songs learned in musical training independently in the kindergartens or at home. When the children saw some music symbols, they would also actively explain the meaning of the symbols to their parents. Music exercises spontaneously carried out by children were very frequent, and parents paid special attention to the music that appeared in their daily life. Miendlarzewska and Trost (2014) conducted a meta-analysis of the influencing factors of musical training, and discovered that genetics, age of musical training onset, motivation to learn music, encouragement from parents/teachers, social development, and the emotional experiences brought about by music all play an important role in the transfer effect of musical training. Children acquire positive emotional experience through musical training, which increases the frequency of spontaneous and independent musical training. This may be one of the reasons why musical training can continue to play a role after it has formally concluded.

In the Digit Span Task, the interaction between measurement time points and groups was significant. The reason for this result may be related to the characteristics of the task itself. Although both the Dot Matrix Test and the Digit Span Task are working memory tasks, the Dot Matrix Test is more of a spatial memory task. Some researchers believe that there is a correlation between music processing and spatial processing. For example, Sluming et al. (2007) examined the behavior and fMRI images of 10 male Orchestra members and 10 matched control subjects while completing a three-dimensional graphic mental rotation task. The results showed that, with the increase in pattern rotation angle (0°, 45°, 90°, 135°, and 180°), the response time of musicians did not change significantly, while that of the control group gradually increased. Further, the correct response rate of musicians in the three-dimensional graphics rotation task was higher than that of participants in the control group. Sluming and other researchers believe that musicians have a stronger ability to sight-play. Sight-playing and spatial processing involve common brain regions (Sluming et al., 2007).

Additionally, long-term training in five-line reading may improve the local processing ability of individuals who have been musically trained. Pietsch and Jansen (2012) found that students majoring in music exhibited higher accuracy in mental rotation processing tasks of three-dimensional graphics than students majoring in education. In the process of musical training, notes are arranged in space, according to certain rules. In the face of a complex arrangement of notes in the five-line staff, musicians tend to process several notes as a group. Therefore, long-term musical training can improve processing of spatial relations. In the present study, children were also trained to read five-line music. 12 weeks after musical training (T3), the training experience related to five-line music reading may have played a role in maintaining spatial memory.

Moreover, to ensure ecological validity, this research had chosen natural classes as training and control groups. Thus, training effects observed occurred in natural environments. EF is more sensitive to environmental stimulation. The experimental group participated in 12 weeks of group musical learning in a regulated and structured environment set by researchers. In this group setting, observational learning likely took place, with the subjects possibly being the trainers (i.e., music teachers) or their peers in the same class. This interchangeable nature of observational learning may amplify the effects of musical training, resulting in the sustained effects observed.

Previous studies have preliminarily explored the core subcomponents of musical training that affect EF (Moreno et al., 2011; Winsler et al., 2011); however, these studies often only examined the influence of musical training on only one subcomponent (Puckering et al., 2014). Therefore, the present study investigated the influence of musical training on three major subcomponents of EF. The results of the current study show that integrated musical training can increase the respective levels of these subcomponents.

### Limitations

There are three main limitations to this study. First, this study used only behavioral experiments to measure the effect of musical training. The influence of musical training on EF is not reflected by activation intensity of a single brain region, but also may be accompanied by changes in the spatial pattern of EF-related brain region activation, as well as the functional linking pattern of related brain regions. Accordingly, future research should not only use ERP, fMRI, and other technologies, but also use multi-modal brain imaging technology to explore the role of musical training on the neural basis of EF. Additionally, EF-related brain structures and functional changes in musical training transfer effects can also be explored. Second, this study tracked the after-effects of musical training. However, the scope of the tracking was limited and only provided a preliminary explanation of the continuous effect of musical training. Finally, we only chose control and experimental groups in this study. In future studies, we will increase the number of different training groups to examine differences among various interventions.

## Conclusion

Results showed that musical training can promote children’s inhibitory control, working memory, and cognitive flexibility. Furthermore, 12 weeks after the experiment, integrated musical training demonstrated a sustained promotion effect. Ultimately, musical training is an appropriate means to promote the development of children’s EF.

## Data Availability

The datasets for this manuscript are not publicly available because the topic is not finished. Requests to access the datasets should be directed to corresponding author.

## Ethics Statement

This study was approved by the local ethics committees of Liaoning Normal University. Written informed consent had been obtained from the parents/legal guardians of all participants.

## Author Contributions

LF and YS designed the experiment. YS, LF, and GL prepared the materials and performed the experiment. YS, LF, YL, and SL analyzed the data and wrote the manuscript.

## Conflict of Interest Statement

The authors declare that the research was conducted in the absence of any commercial or financial relationships that could be construed as a potential conflict of interest.
